# Temperature dependence of intrinsic and extrinsic contributions to anisotropic magnetoresistance

**DOI:** 10.1038/s41598-021-00374-8

**Published:** 2021-10-22

**Authors:** Ji-Ho Park, Hye-Won Ko, Jeong-Mok Kim, Jungmin Park, Seung-Young Park, Younghun Jo, Byong-Guk Park, Se Kwon Kim, Kyung-Jin Lee, Kab-Jin Kim

**Affiliations:** 1 Department of Physics, KAIST, Daejeon, 34141 South Korea; 2 Department of Materials Science and Engineering and KI for Nanocentury, KAIST, Daejeon, 34141 South Korea; 3 Division of Scientific Instrumentation & Management, KBSI, Daejeon, 34133 South Korea

**Keywords:** Materials science, Physics

## Abstract

Electrical conduction in magnetic materials depends on their magnetization configuration, resulting in various magnetoresistances (MRs). The microscopic mechanisms of MR have so far been attributed to either an intrinsic or extrinsic origin, yet the contribution and temperature dependence of either origin has remained elusive due to experimental limitations. In this study, we independently probed the intrinsic and extrinsic contributions to the anisotropic MR (AMR) of a permalloy film at varying temperatures using temperature-variable terahertz time-domain spectroscopy. The AMR induced by the scattering-independent intrinsic origin was observed to be approximately 1.5% at *T* = 16 K and is virtually independent of temperature. In contrast, the AMR induced by the scattering-dependent extrinsic contribution was approximately 3% at *T* = 16 K but decreased to 1.5% at *T* = 155 K, which is the maximum temperature at which the AMR can be resolved using THz measurements. Our results experimentally quantify the temperature-dependent intrinsic and extrinsic contributions to AMR, which can stimulate further theoretical research to aid the fundamental understanding of AMR.

## Introduction

Electrical conduction in magnetic metals depends on their magnetic state, which results in various magnetoresistances (MRs), such as anisotropic MR (AMR)^1^, giant MR (GMR)^2,3^, magnon MR (MMR)^4^, and spin Hall MR (SMR)^5^ as well as various Hall effects^6,7^. The microscopic mechanisms of such MRs have been extensively studied and have so far been classified into either intrinsic or extrinsic origins^6,7^.

Among the various MRs, the AMR is one of the most fundamental spin–orbit–interaction (SOI)-induced transport phenomena in magnetic materials. The AMR describes the anisotropic charge conductivity and its dependence on the relative orientation of the current flow and the magnetization. Early theories viewed the AMR through Mott’s two-current model^8^, in which the electrical conduction in transition metals is modelled as the sum of two separate currents of majority and minority spin electrons, with the following two assumptions: (i) *s* electrons are responsible for the conduction, owing to the relatively low mobility of *d* electrons; (ii) the large density of *d* states mainly accounts for the scattering rates of *s* electrons. This led to AMR being attributed to the magnetization-dependent scattering time, i.e., the extrinsic effect^9–13^, as the *d* electrons experience the SOI but the *s* electrons do not. Specifically, the conducting *s* electrons experience anisotropic scattering processes via spin–orbit-coupled *d* bands. This class of AMR theories take into account various SOI-induced effects on the scattering time, such as the mixing of majority and minority *d* states^11–13^, altering the atomic wave function of *d* orbitals^12^, and spin mixing processes including spin-flip scattering^10^.

Recent theories have diverted from the assumptions of the Mott model and have identified the intrinsic mechanism of AMR, which arises from the scattering-independent band-structure effect. The resulting intrinsic relaxation is essential for transition metals since *d* electrons also participate in electrical conduction^14,15^ through *spd* hybridization^16^. In other words, the conduction electrons are no longer intact to SOI; rather, their band structures are affected by SOI. Therefore, not only does the magnetization-dependent scattering time play a crucial role in the AMR, but the magnetization-dependent electronic structure, i.e., the intrinsic origin, is also critical. Recent theoretical works have emphasized the intrinsic effect on AMR by illustrating ballistic AMR owing to the anisotropic *d* bands^17^ and demonstrating the magnetization-dependent band structure owing to the orbital hybridization^18^. The existence of the intrinsic contribution has also been investigated experimentally. Hupfauer et al. demonstrated the crystalline AMR effect, originating from the difference in electronic density, through its magnetization orientation^19^, and Zeng et al. reported the intrinsic contribution caused by the magnetization-direction-dependent band crossing effect^20^.

Recently, Nadvornik et al. have successfully disentangled the intrinsic and extrinsic contributions to AMR in polycrystalline thin films at room temperature^21^, in which they found that the anisotropy of a crystal structure could be an origin of intrinsic contribution to AMR. Despite the success, however, our understanding of AMR is still far from satisfactory. In particular, the temperature dependence of the intrinsic and extrinsic contributions to AMR remains elusive, despite the large temperature variance of AMR^22^. In this respect, investigating the temperature dependence of the intrinsic and extrinsic contributions to AMR is of crucial importance to understand the fundamental origins of the AMR.

In this letter, we investigate the temperature dependence of the intrinsic and extrinsic contributions to AMR in a permalloy film by using terahertz time-domain spectroscopy (THz-TDS). We find that the intrinsic contribution to AMR was virtually independent of temperature, while the extrinsic contribution to AMR decreased with increasing temperature, suggesting that the portion of intrinsic contribution to the total AMR increases with increasing temperature. Quantitatively, the intrinsic portion accounted for $(32.3\pm 7.3)$% of the total AMR at *T* = 16 K, which increased to ($46.2\pm 9.7)$% at *T* = 155 K. Our results provide experimental evidence for the explicit temperature dependence of the intrinsic and extrinsic contributions, which can stimulate further theoretical research towards a comprehensive understanding of AMR.

## Results

### Identifying the intrinsic and extrinsic contribution based on the Drude model

As the AMR stems from changes in the longitudinal conductivity, the DC Drude model^23^ can be used to investigate the intrinsic and extrinsic contributions to the AMR:

1 $$\sigma_{dc} = \frac{n}{{m^{*} }}e^{2} \tau ,$$

where ${\sigma }_{dc}$ is the DC conductivity, $n$ is the charge density, ${m}^{*}$ is the effective mass, $e$ is the electron charge, and $\tau$ is the momentum scattering time. Here, the magnetization-dependent change in $\frac{n}{{m}^{*}}$ and $\tau$ represent the scattering-independent intrinsic and scattering-dependent extrinsic contributions, respectively^21^. Therefore, the intrinsic and extrinsic contributions to the AMR can be separately identified by measuring both $\frac{n}{{m}^{*}}$ and $\tau$ for different magnetization directions. We note that the Drude model can be applied even in the presence of *spd* hybridization, because the conduction of *d*-electrons can be neglected due to their localized characteristics and large effective mass^24^.

The independent probing of $\frac{n}{{m}^{*}}$ and $\tau$ can be achieved by measuring the AC Drude conductivity with the THz-TDS. The AC Drude conductivity, $\stackrel{\sim }{\sigma }(\omega )$, is given by

2 $$\tilde{\sigma }\left( \omega \right) = \frac{{\sigma_{dc} }}{1 - i\omega \tau } = \frac{{\sigma_{dc} }}{{1 + \omega^{2} \tau^{2} }} + i\omega \tau \frac{{\sigma_{dc} }}{{1 + \omega^{2} \tau^{2} }},$$

where $\omega /2\pi$ is the THz frequency. THz-TDS allows us to determine the real and imaginary components of the AC Drude conductivity, leading to the direct determination of the momentum scattering time, $\tau$, and the DC conductivity, ${\sigma }_{dc}$, simultaneously, based on Eq. (2). Therefore, it enables the separate quantification of $\tau$ and $\frac{n}{{m}^{*}}$ through using Eq. (1), which correspond to the extrinsic and intrinsic contributions to AMR, respectively.

### Disentangling the intrinsic and extrinsic contributions using THz-TDS

**Figure 1:**
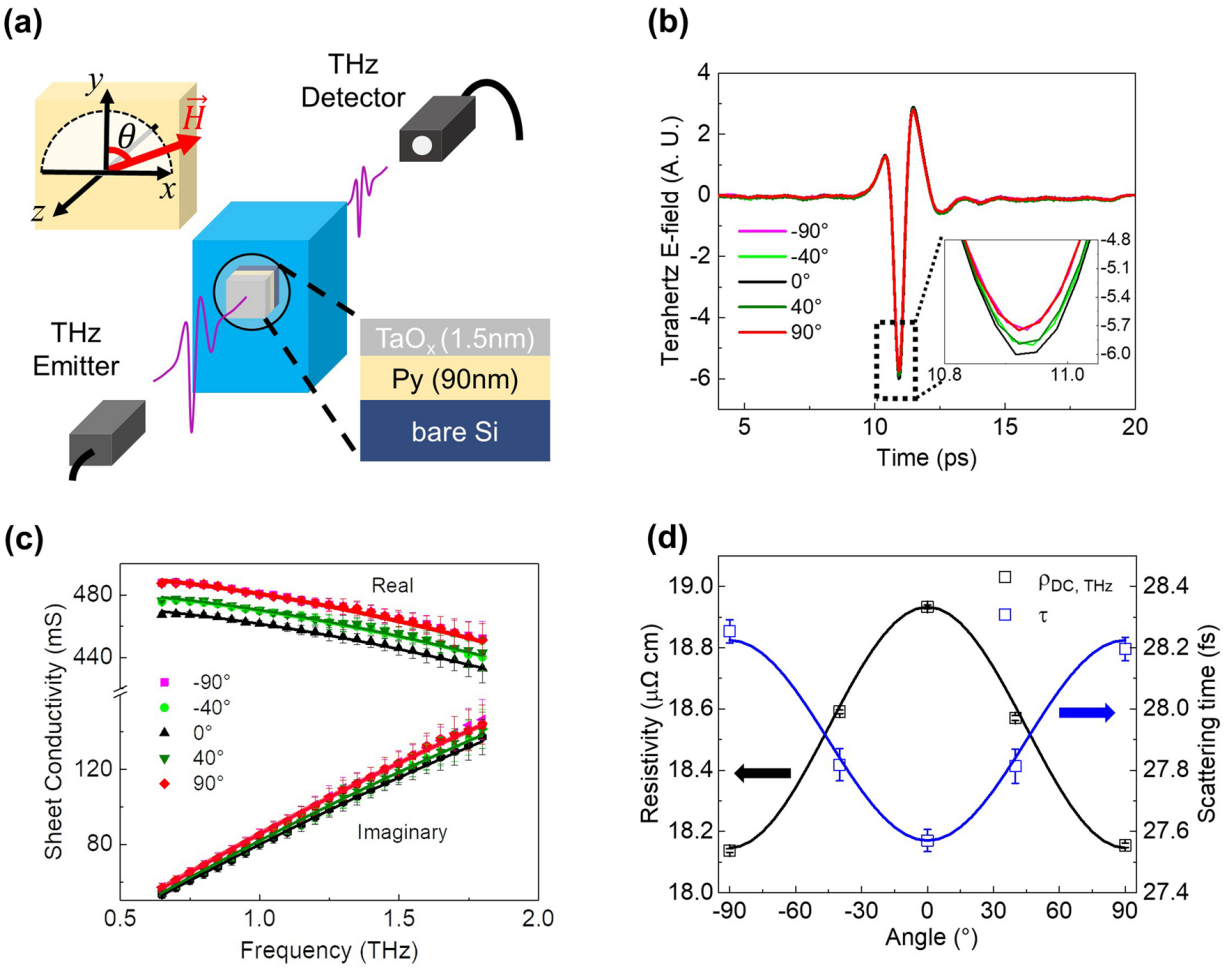
Angle-dependent complex conductivity measurement. (**a**) Schematic illustration of terahertz time-domain spectroscopy (THz-TDS). (**b**) Time-domain THz pulses for several angles between THz electric field and magnetization of sample. Inset zooms into the amplitude of transmitted pulses. The measurement temperature is *T* = 54 K. (**c**) The real and imaginary parts of complex conductivity with respect to the THz frequency for several angles. Solid lines are the Drude fittings using Eq. (2). (**d**) Angle-dependent DC resistivity (black) and scattering time (blue) determined by THz spectroscopy. The error bars for DC resistivity are smaller than the symbol size.

Figure 1a shows the schematic of our THz-TDS setup. The 90 nm-thick permalloy (Py) film, deposited on an Si substrate by magnetron sputtering, was located inside the cryostat. The weak single-cycle, sub-picosecond THz pulse was directed normally at the sample (along the *z*-axis) and its transmission was measured. The polarization of the THz electric field lies along the *y*-axis (i.e., $\theta =0$), and the magnetization direction of the Py was controlled in the *x*–*y* plane (with an angle $\theta$) by applying an in-plane magnetic field using a home-built vector magnet (see “Methods” for the sample fabrication and THz-TDS setup).

Figure 1b shows the typical transmitted THz time-domain signal obtained upon varying the angle $\theta$ between the THz electric field and Py magnetization. We applied a magnetic field of 25 mT, which is large enough to saturate the magnetization of the Py film. The measurement was conducted at *T* = 54 K. To increase the signal to noise ratio, we averaged the time-domain trace by accumulating 850 pulses for each measurement, and this measurement was repeated 40 times (see “Methods” for measurement details). As the oscillating THz electric field induces a time-dependent current in the sample, the attenuation and phase delay of THz electric field can be observed as it propagates through the sample. The degree of attenuation depends on the DC resistivity of the sample: the higher the resistivity, the smaller the attenuation. Since the Py has a positive AMR^25^, it is noted that ${\rho }^{AMR}\equiv \frac{{\rho }^{\parallel }-{\rho }^{\perp }}{{\rho }^{\perp }}>0$, where ${\rho }^{\parallel }$(${\rho }^{\perp }$) is the resistivity of the Py when the current and magnetization are parallel (perpendicular), and that the resistivity of the sample is high (low) when the magnetization and the electric current are parallel (perpendicular). Therefore, a large (small) attenuation of the THz electric field is expected when the THz electric field and the Py magnetization are perpendicular (parallel) to each other. This is indeed observed in our THz time-domain signals in Fig. 1b.

To extract the intrinsic and extrinsic parameters, we obtained the complex conductivity of the sample by performing a Fourier transform on the THz time-domain signals (see “Methods” for details). Figure 1c shows the THz spectra of the real and imaginary parts of the complex conductivity for 5 different angles ($\theta$= 90°, 40°, 0°, − 40°, − 90°). The solid lines are the best fits using the AC Drude model based on Eq. (2). The correlation of the experimental data and the AC Drude fitting demonstrates that the complex conductivity of Py is well described by the AC Drude model. The real and imaginary components of the complex conductivities directly yield the momentum scattering time, $\tau$, and the DC resistivity $\left({\rho }_{dc,THz}=\frac{1}{{\sigma }_{dc,THz}}\right)$ simultaneously through using Eq. (2). We plot those values in Fig. 1d. Both $\tau$ (blue symbols) and ${\rho }_{dc,THz}$ (black symbols) follow the typical AMR angle dependence (~ ${\mathrm{cos}}^{2}\theta$, shown by the solid lines in Fig. 1d) and are inversely proportional to each other in accordance with $\frac{1}{{\rho }_{dc,THz}}={\sigma }_{dc,THz}=\frac{n}{{m}^{*}}{e}^{2}\tau$. However, we found that the amount of anisotropy was different for ${\rho }_{dc,THz}$ (4.3%) than that for $\tau$ (2.8%), which implies a finite intrinsic contribution originated from $n/{m}^{*}$.

### Temperature dependence of transport parameters

**Figure 2:**
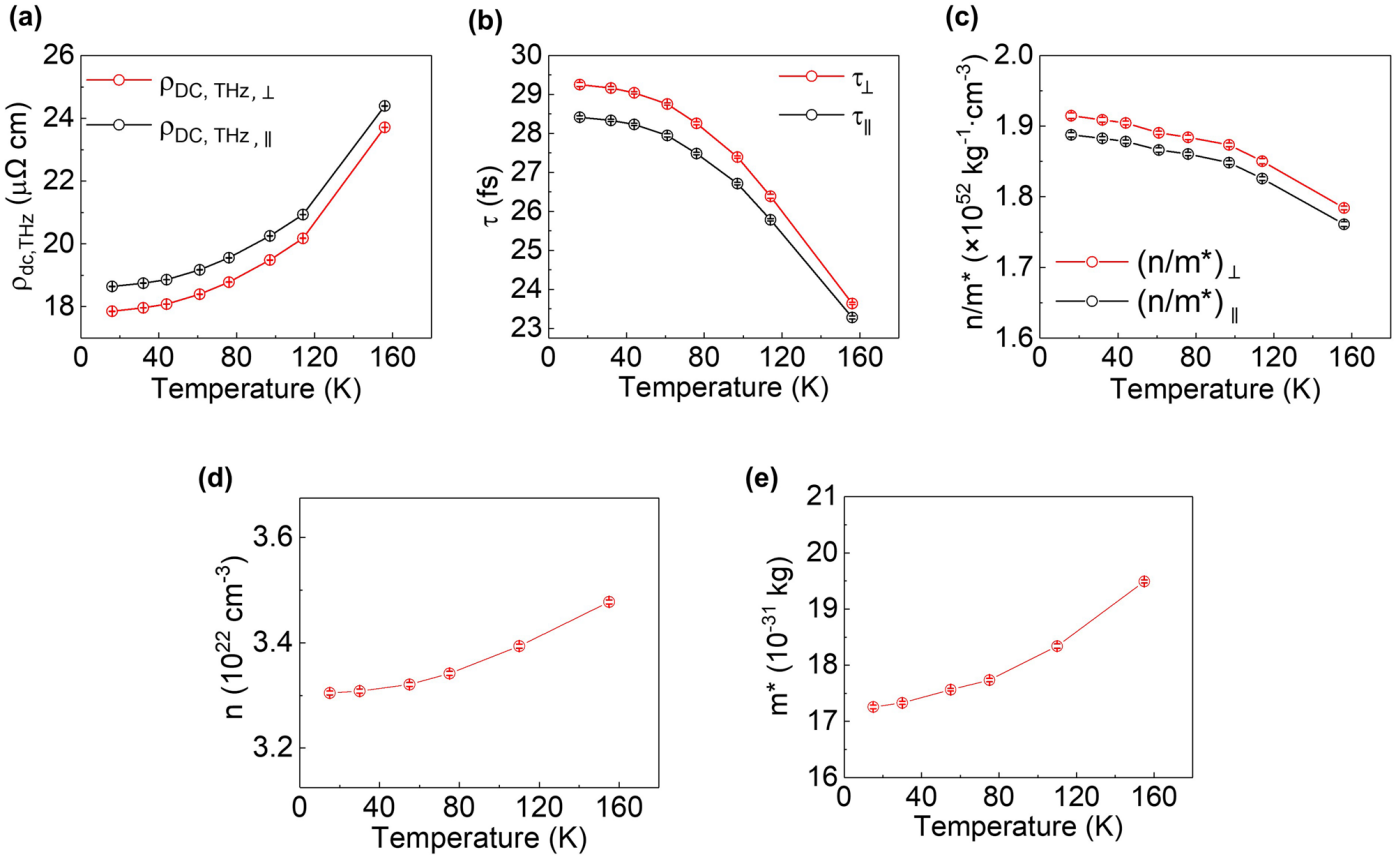
Temperature-dependent intrinsic and extrinsic transport parameters probed by THz-TDS. (**a**) THz-proved DC resistivity, ${\rho }_{dc, THz}$, as a function of temperature for parallel (black) and perpendicular (red) geometries. (**b**) Temperature-dependent electron scattering time, $\tau$, for parallel (black) and perpendicular (red) geometries. (**c**) Temperature-dependent charge density/effective mass, $n/{m}^{*}$, for parallel (black) and perpendicular (red) geometries. (**d**) Charge concentration, $n$, as a function of temperature obtained from ordinary Hall effect measurement. (**e**) effective mass,${m}^{*}$, as a function of temperature. The error bars in (**a**)–(**e**) are smaller than the symbol size.

We next investigated the temperature dependence of intrinsic and extrinsic transport parameters by repeating the experiment at various temperatures. For accuracy, we performed THz-TDS measurements for both parallel and perpendicular geometries 160 times at a fixed temperature (see Methods and Supplementary Note 1 for details of measurement). Figure 2a–c show the temperature dependence of ${\rho }_{dc, THz}$ [Fig. 2a], $\tau$ [Fig. 2b], and $\frac{n}{{m}^{*}}$ [Fig. 2c] for parallel (black) and perpendicular (red) geometries. Here, ${\rho }_{dc, THz}$ and $\tau$ were directly determined from the THz-TDS measurements and $\frac{n}{{m}^{*}}$ extracted using Eq. (1) and ${\rho }_{dc, THz}$ and $\tau$ measurements. Figure 2a,b show that as the temperature increased, ${\rho }_{dc, THz}$ increased but $\tau$ decreased. This follows the typical trends of metallic samples, which originate from the increased effect of electron scattering by thermally excited phonons and magnons^26^. The temperature dependence of ${\rho }_{dc, THz}$ is further confirmed by the DC resistivity measured separately [see Supplementary Note 2]. We note that the measurement temperature was limited to *T* < 155 K where the Drude fitting was guaranteed; at higher temperatures, the uncertainty in determining $\tau$ increases due to the rapid decrease of $\tau$, causing the error bars of $\tau$ for parallel and perpendicular geometries to overlap.

Figure 2c shows that not only the scattering-dependent $\tau$ but also the scattering-independent $\frac{n}{{m}^{*}}$ varied with temperature. For further in-depth quantitative analysis, we separately determined the carrier density, $n$, by measuring the ordinary Hall effect. Figure 2d shows the temperature dependence of $n$, which increased by approximately 5% when the temperature increased from *T* = 16 K to *T* = 155 K. The temperature dependence of ${m}^{*}$ could then be extracted from the measured ${(n/{m}^{*})}_{\perp}$ [Fig. 2c] and $n$ [Fig. 2d]. Here, we used ${(n/{m}^{*})}_{\perp}$ for consistency as the Hall effect was measured within this perpendicular geometry. Figure 2e shows that ${m}^{*}$ increased by approximately 13% when the temperature is increased from *T* = 16 K to *T* = 155 K. These results suggest that both $n$ and ${m}^{*}$, which constitute the intrinsic contribution to AMR, vary with temperature. We note that the values of $n$ and ${m}^{*}$ as well as their temperature dependences are consistent with those reported previously^27–32^.

### Temperature dependence of intrinsic and extrinsic contributions to AMR

**Figure 3:**
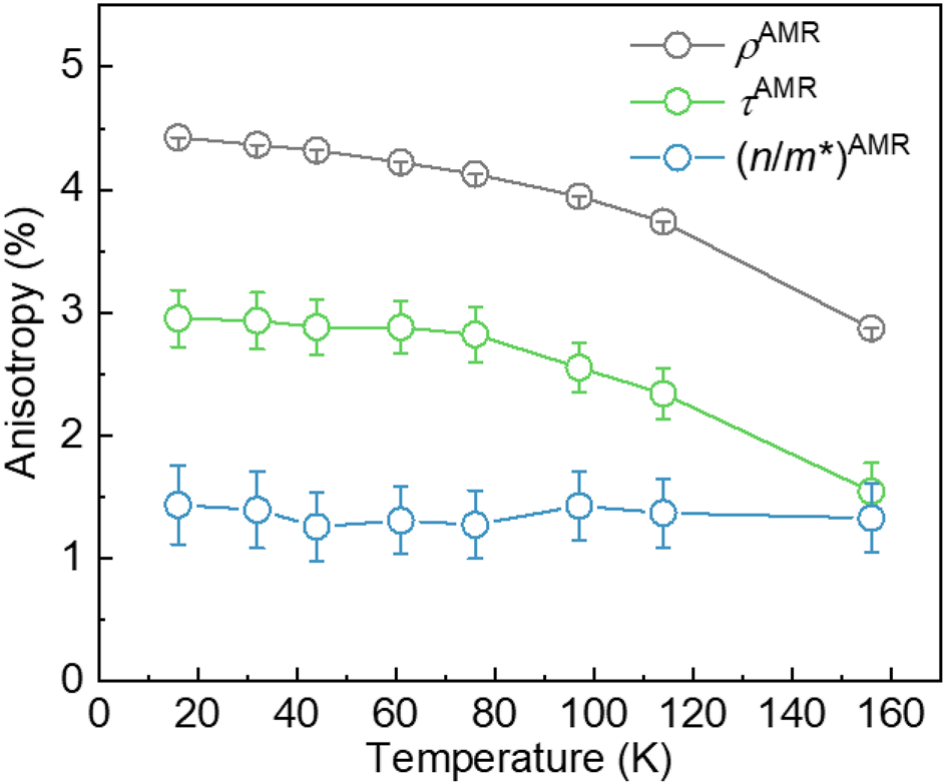
Intrinsic and extrinsic contributions to total AMR. Anisotropy ratios of $${\rho }_{dc, THz}$$ (gray), $$\tau$$ (green) and ($$n/{m}^{*})$$ (blue) as a function of the temperature. Here, $${\rho }^{AMR}$$ corresponds to the total AMR, while $${\tau }^{AMR}$$ and $${(n/{m}^{*})}^{AMR}$$ represent the extrinsic and intrinsic contribution to AMR. The error bars for $${\rho }^{AMR}$$ are smaller than the symbol size.

The clear difference between the results obtained with the parallel and perpendicular geometries in Fig. 2a–c indicates that all parameters are anisotropic and thus contribute to the AMR. In Fig. 3, we summarize the temperature dependent anisotropy for ${\rho }^{AMR}\equiv \frac{{\rho }_{dc,THz,\parallel} \; - {\rho }_{dc,THz,\perp }}{{\rho }_{dc,THz,\perp }}$ [grey] and ${\tau }^{AMR}\equiv \frac{{\tau }_{\perp }-{\tau }_{\parallel }}{{\tau }_{\parallel }}$ [green] as well as for ${\left(n/{m}^{*}\right)}^{AMR}\equiv \frac{{\left(n/{m}^{*}\right)}_{\perp }-{\left(n/{m}^{*}\right)}_{\parallel }}{{\left(n/{m}^{*}\right)}_{\parallel }}$ [blue], which have been extracted from Fig. 2a–c. It is clear that not only the scattering-dependent extrinsic contribution $\tau$ but also the scattering-independent intrinsic contribution $n/{m}^{*}$ constitutes the AMR [the origin of the intrinsic contribution is discussed in Supplementary Note 3]. The temperature dependence of the extrinsic and intrinsic contributions show distinctive behaviour: the ${\tau }^{AMR}$ largely decreases with increasing temperature, even when taking the error bars into consideration [green symbols in Fig. 3], while ${\left(n/{m}^{*}\right)}^{AMR}$ does not exhibit a clear change but is still within the range of its error bars [blue symbols in Fig. 3]. Quantitatively, ${\tau }^{AMR}$ is approximately 3.0% at *T* = 16 K but decreases to 1.5% at *T* = 155 K, corresponding to a decrease by half as the temperature increases from *T* = 16 K to *T* = 155 K. In contrast, ${n/{m}^{*}}^{AMR}$ is approximately 1.5% at *T* = 16 K and remains virtually constant up to *T* = 155 K. This means that the portion of the intrinsic contribution to the total AMR gradually increases with increasing temperature, mainly due to the reduction of extrinsic contributions at higher temperature. The intrinsic portion is ($32.3\pm 7.3)$% of the total AMR at *T* = 16 K, but it increases ($46.2\pm 9.7)$% at *T* = 155 K.

## Conclusion

We investigated the temperature dependence of intrinsic and extrinsic contributions to the AMR in a Py film by independently probing the electron-momentum scattering time ($\tau$) and fraction of charge density to effective mass ($n/{m}^{*}$) at various temperatures using THz-TDS. The intrinsic contribution was approximately 1.5% at *T* = 16 K and was virtually independent of temperature, while the extrinsic contribution was approximately 3.0% at *T* = 16 K and decreased by half as the temperature increased up to *T* = 155 K. Our results therefore provide experimental evidence for the distinctive temperature dependence of the intrinsic and extrinsic AMR and call for further theoretical investigations to enable a comprehensive understanding of AMR accounting for the intrinsic and extrinsic mechanisms. Finally, we anticipate that the use of THz-TDS in the various MRs, especially for MR having distinct temperature dependence, will lead to substantial advances in our understanding on the fundamentals of spintronic transport.

## Methods

### Sample preparation

90-nm-thick Py films with 1.5 nm TaO_x_ capping layer were deposited by dc magnetron sputtering on Si substrate. The Py films had in-plane magnetic anisotropy, and the size of films was 6 × 12 mm^2^ which was larger than the diffraction limit of THz wave (3 mm). To measure the complex conductivity of sample, we prepared bare Si substrate as a reference which has almost identical thickness with that of the deposited sample. For this study, we prepared three Py films (#1—#3): #1 was used for angle dependent THz-TDS measurement (Fig. 1), #2 was used for temperature dependent THz-TDS measurement (Figs. 2 and 3), and #3 was used for four-probe DC resistance measurement (Fig. S2). Nominal thickness of samples was same for all samples.

### Experimental setup for THz-TDS

A standard THz-TDS setup (Tera K15-Menlosystems) with 4 TPX lenses was used for THz measurement. Montana vacuum cryostat was used to control the temperature of sample. A standard glass windows were replaced with the TPX windows for THz experiment. The deposited film and reference substrate were installed simultaneously inside the cryostat using L-shaped sample holder. Two samples (deposited film and reference substrate) were attached to each plane of L-shaped holder that was attached at the ANC300 piezo rotator. By rotating the piezo rotator, we could selectively choose the deposited film or reference substrate without breaking the vacuum. An in-plane magnetic field was applied by using home-made vector electromagnet. The vector magnet is made of two axis electromagnets, each of which generates magnetic field of up to 100 mT. The directional magnetic field can be applied by adjusting the strengths of two orthogonal magnetic fields. The direction of the magnetic field is confirmed by sensing the magnetic field at sample position using Gaussmeter. To rule out any mechanical effects, every structure inside the vacuum chamber is made of OFHC (Oxygen free high thermal conductivity) copper and brass which have very low magnetic susceptibility. Field-induced mechanical effects have been confirmed to be negligibly small (see Supplementary Note 4).

### THz measurement for AMR

A single cycle THz pulse with 2 ps duration was generated from the THz emitter and then, the THz beam was focused on sample through the TPX lens. The transmitted pulse was collimated and detected at the THz detector. The time domain data was obtained by the pump-probe method with 0.033 ps time interval. To acquire the AMR, each time-domain data was averaged by accumulating 850 repeated measurements, and this THz measurement was repeated up to 160 times (40 times for Fig. 1) for parallel and perpendicular geometries, respectively, yielding the total average number of 136,000 (34,000 for Fig. 1). To reduce the noise, we performed the following treatments: (1) THz system was purged with dry nitrogen gas until the relative humidity was down to less than 1%. (2) A 675 $\mathrm{\mu m}$ thick substrate was chosen to keep internally reflected pulse away from the main pulse. (3) Time domain was carefully selected in order to exclude the effect of internal reflections which would cause the ripple in the frequency domain. (4) High resistive Si was used to reduce the absorption loss in substrate. (5) Both sides of substrate were polished to reduce the effect from surface roughness which would affects the transmittance of THz pulse. (6) The thickness of Py (90 nm) was chosen to increase the difference of the sheet conductivity without significantly deteriorating the signal-to-noise ratio.

### Complex conductivity measurement procedure

To obtain complex sheet conductivity, the detected time domain signal was first converted to the frequency domain by Fourier transform (see Supplementary 5), which provides the frequency-dependent amplitude $A(\upomega )$ and phase $\phi (\upomega )$ of electric field $E\left(\omega \right)$. This procedure was repeated for reference substrate, and obtained ${A}_{\mathrm{ref}}(\upomega )$ and ${\phi }_{\mathrm{ref}}(\upomega )$ of ${E}_{\mathrm{ref}}\left(\omega \right)$. The obtained amplitudes and phases were then inserted in the following Tinkham equation^33^

3 $$\frac{E\left( \omega \right)}{{E_{{{\text{ref}}}} \left( \omega \right)}} = \frac{{n_{sub} + 1}}{{n_{sub} + 1 + Z_{0} \tilde{\sigma }_{s} \left( \omega \right)}},$$

where ${Z}_{0}$ is the vacuum impedance and ${n}_{sub}$ is the refractive index of substrate. By inserting the experimentally obtained $E\left(\omega \right)=A\left(\omega \right){e}^{i\phi \left(\omega \right)}$ and ${E}_{\mathrm{ref}}\left(\omega \right)={\mathrm{A}}_{\mathrm{ref}}\left(\omega \right){e}^{i{\phi }_{\mathrm{ref}}\left(\omega \right)}$ into Eq. (3), the complex conductivity reads

4 $$\begin{aligned} \widetilde{{\sigma_{s} }}\left( \omega \right) & = \left[ {\left( {\frac{{{\text{A}}_{{{\text{ref}}}} \left( \omega \right)e^{{i\phi_{{{\text{ref}}}} \left( \omega \right)}} }}{{A\left( \omega \right)e^{i\phi \left( \omega \right)} }}} \right) - 1} \right]*\frac{{\left[ {n_{sub} + 1} \right]}}{{Z_{0} }} \\ & = \left[ {\left\{ {\frac{{{\text{A}}_{{{\text{ref}}}} \left( \omega \right)}}{A\left( \omega \right)}cos\left( {\phi_{{{\text{ref}}}} \left( \omega \right) - \phi \left( \omega \right)} \right) - 1} \right\} + i\left\{ {\frac{{{\text{A}}_{{{\text{ref}}}} \left( \omega \right)}}{A\left( \omega \right)}sin\left( {\phi_{{{\text{ref}}}} \left( \omega \right) - \phi \left( \omega \right)} \right)} \right\}} \right]*\frac{{\left[ {n_{sub} + 1} \right]}}{{Z_{0} }}, \\ \end{aligned}$$

Then one can obtain the real and imaginary parts of complex sheet conductivity ${\stackrel{\sim }{\sigma }}_{s}$. Here, the refractive index of substrate was obtained by independent experiment^34^. Once the real and imaginary conductivities were obtained, the scattering time and DC conductivity can be directly determined by using Eq. (2) in the main manuscript without any assumptions on charge density and effective mass.

### Reliability of fitting

For Drude fit, we used “instrumental error weighting function” which gives weight to each data inversely proportional to the size of error (that is, data point with smaller error would affect more). The error bars in Figs. 2 and 3 in the main manuscript were the results from the fitting based on the “instrumental error weighting function”. We note that the fitting error does not much vary even when we use a “standard equal weighting function”, since our complex conductivity data have very small error (the change in $\tau$ depending on the fitting function is about 0.03%). To check the effect of fitting frequency range, we fitted the data by reducing the frequency range to 1 THz < *f* < 1.5 THz, instead of 0.75 THz < *f* < 1.75 THz. The resulting $\tau$ does not change within 0.04%. Considering that the error in $\tau$ is about 0.2% in our result, the selection of fitting function as well as the fitting frequency range does not much affect the determination of $\tau$.

### Determination of error bars

For the case of experimental data (Fig. 1c and Fig. S1), error bars were defined as 1 $\upsigma$ of iterative THz conductivity measurements (40 times repeated for Fig. 1c and 160 times repeated for Fig. S1). Real and imaginary part of complex conductivities were fitted by AC Drude model based on Kramer – Kronig relation. In other words, they were simultaneously fitted. Error bars in fitted data (AC Drude fitting for Figs. 1d,2a,b and Fig. S2, Linear fitting for Fig. 2d) were fitting error with instrumental error weighting. As we described in Methods and Supplementary Note 5, since our data do not scattered around fitting line, choice of error weighting is not important and size of fitting error bars could be small. Lastly, error bars in Figs. 2c,e,3 (data obtained by subtracting or dividing fitted values) were determined by summing all errors of parameters that used for subtracting or dividing.

## Supplementary Information

Supplementary Information.
