# Supplementary material for: Temperature dependence of intrinsic and extrinsic contributions to anisotropic magnetoresistance
Source: Sci Rep. 2021 Oct 22;11:20884. doi: 10.1038/s41598-021-00374-8 (PMC8536661; doi:10.1038/s41598-021-00374-8)
Supplement: Supplementary file 1 — Supplementary Information. [file 41598_2021_374_MOESM1_ESM.docx]

Supplementary Note for

“Temperature dependence of intrinsic and extrinsic contributions to anisotropic magnetoresistance”

Ji-Ho Park^1^, Hye-Won Ko^1^, Jeong-Mok Kim^2^, Jungmin Park^3^, Seung-Young Park^3^, Younghun Jo^3^, Byong-Guk Park^2^, Se Kwon Kim^1^, Kyung-Jin Lee^1^, and Kab-Jin Kim^1★^

*^1^Department of Physics, KAIST, Daejeon 34141, South Korea*

*^2^Department of Materials Science and Engineering and KI for Nanocentury, KAIST, Daejeon 34141, South Korea*

*^3^Center for Scientific Instrumentation, KBSI, Daejeon 34133, South Korea*

*South Korea*

**-Contents-**

**Note 1. Real and imaginary conductivity data for various temperatures**

**Note 2. DC resistivity by four-probe measurement**

**Note 3. Existence of intrinsic AMR in polycrystalline films**

**Note 4. Possible field-induced artefacts**

**Note 5. Fourier transform of time domain pulse**

**Note 1. Real and imaginary conductivity data for various temperatures**

Figure S1 shows the raw data sets for Fig. 2 in the main manuscript, which exhibit the real and imaginary spectra for various temperatures. One can notice that the error bars in Fig. S1 are smaller than those in Fig. 1(c) due to the large average number (34,000 for Fig. 1(c) and 136,000 for Fig. S1).


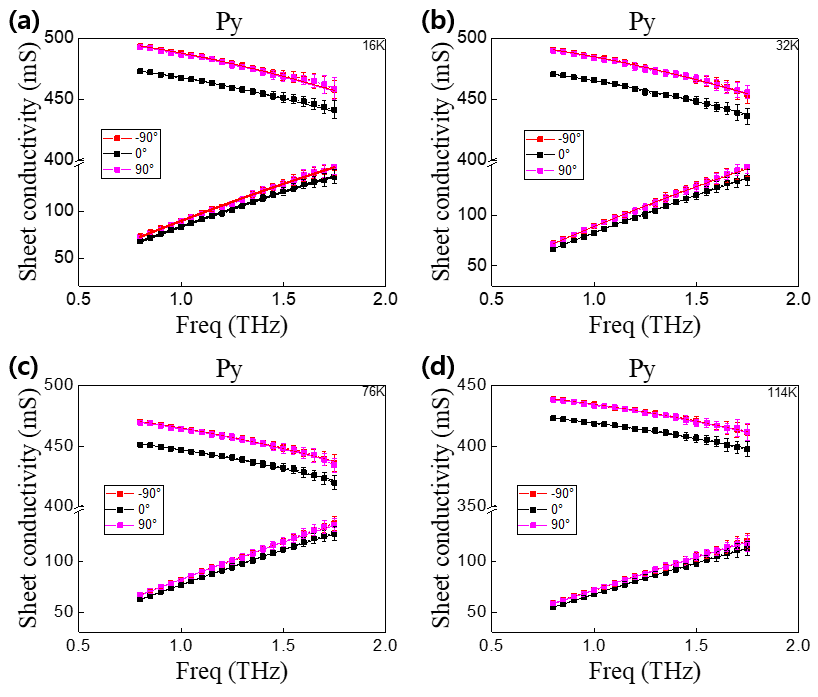


**Fig. S1** The real and imaginary parts of complex conductivity with respect to the THz frequency for several angles. Solid lines are the AC Drude fittings. (a) 16K (b) 32K (c) 76K (d) 114K.

**Note 2. DC resistivity by four-probe measurement**

We performed the standard DC resistivity measurement using Physical Property Measurement System (PPMS) at various temperatures. The obtained DC resistivity are plotted in Fig. S2 as closed symbols. The result shows that the resistivity decreases with decreasing temperature, signalling the suppression of electron scattering by thermally activated phonons/magnons. Notably, the resistivity saturates for *T* < 20 K, which implies that the resistivity for *T* < 20 K is mainly affected by the scattering of electron by quenched impurities.

We then compared the DC resistivity obtained by PPMS (closed symbols in Fig. S2) with that obtained by THz-TDS (open symbols in Fig. S2). Two DC resistivities obtained from different methods are consistent with each other, confirming the reliability of our measurement and fitting. The slight difference between standard four-probe DC measurement and THz-TDS is possibly due to the sample differences.


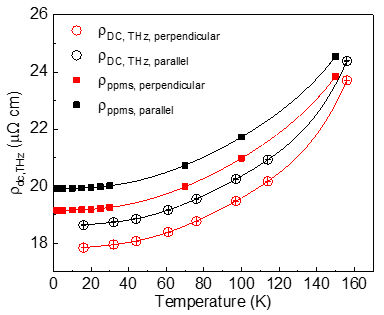


**Fig. S2** Temperature dependent resistivity for parallel and perpendicular geometries of Py (90nm) sample by THz-TDS (open symbols) and standard four-probe DC measurements done by PPMS (closed symbols).

**Note 3. Existence of intrinsic AMR in polycrystalline films**

The intrinsic AMR can exist even in the polycrystalline films as follows. In polycrystalline samples, where the crystalline axis is randomly distributed among grains, the dependence of the carrier density $n$ and the effective mass $m^{*}$ on the wavevector $\boldsymbol{k}$ and the magnetization direction $\boldsymbol{m}$ can be phenomenologically written as $n\left( \boldsymbol{k};\boldsymbol{m} \right)=n\left( \boldsymbol{k}\cdot\boldsymbol{m} \right)$ and $m^{*}\left( \boldsymbol{k};\boldsymbol{m} \right)=m^{*}\left( \boldsymbol{k}\cdot\boldsymbol{m} \right)$, where $\boldsymbol{k}$ and $\boldsymbol{m}$ appear only through their inner product (since there is no preferential direction due to the polycrystalline nature). If the inversion symmetry is not broken macroscopically in polycrystalline samples, we have more stringent functional dependence: $n\left( \boldsymbol{k};\boldsymbol{m} \right)=n\left( \left( \boldsymbol{k}\cdot\boldsymbol{m} \right)^{2} \right)$ and $m^{*}\left( \boldsymbol{k};\boldsymbol{m} \right)=m^{*}\left( \left( \boldsymbol{k}\cdot\boldsymbol{m} \right)^{2} \right)$, which gives rise to the anisotropy that is discussed in our manuscript. These expressions meet all the symmetry properties required by polycrystalline ferromagnetic samples. Therefore, a certain amount of anisotropy in $n/{m^{*}}$ is expected to occur even in polycrystalline ferromagnets.

Although the anisotropy in $n/{m^{*}}$ would be larger in single-crystal samples than in polycrystalline samples, as argued above, even in polycrystalline films, the finite anisotropy is expected to exist in $n/{m^{*}}$, meaning that the change of the band structure due to the external field via spin-orbit coupling gives rise to a finite anisotropy in $n/{m^{*}}$ even after being averaged among grains with different crystal orientations. We note that similar argument has been employed to show the existence of the intrinsic anomalous Hall effect in polycrystalline films, where the angular average of intrinsic contributions for all crystalline directions was found to be finite in good agreement with the measured intrinsic contribution for polycrystalline films [35].

In polycrystalline metals, there are two important length scales, the electron mean free path and the average grain size, within which the electronic band structure is well defined locally. It is reasonable to classify the contributions to AMR from the anisotropy of $n/{m^{*}}$as intrinsic ones if the grain size is larger than the mean free path so that electron dynamics can manifest the local band structure. In other words, as long as the electrons feel the band structure of each grain, we expect that there is a finite (average) intrinsic contribution to AMR.

To further support our claim, we have checked the microstructure of our Py film by X-ray diffraction (XRD) measurement. Figure S3 shows the XRD spectra for our polycrystalline Py film. A peak was clearly observed at $2\theta\sim44^{\circ}$ in the spectra, indicating the fcc (111) crystalline phase of Py [36]. The grain size can be estimated from the spectra based on the Scherrer formula, and we found that the average grain size was about 12.8 nm. As the grain size is larger than the electron mean free path of about 6 nm in Py, one can expect that the electrons feel the band structure of each grain, which could give rise to the average intrinsic contribution (Here, we estimated the electron mean free path from the Fermi velocity [37] ($v_{F}=0.22\times{10}^{6} m/s$) and measured scattering time ($\tau=23\sim30 fs$ in Fig. 2(b) in our manuscript)).

Despite the discussion above, the existence of intrinsic contribution in Py films needs to be further clarified, because the recent work claimed that the AMR of Py films is of predominantly extrinsic nature although they measured at room temperature [21]. The different results may be due to the different thickness, which could result in interface-dominant transport at thinner layer (8 nm in ref. [21]) or bulk-dominant transport at thicker layer (90 nm in our work) [38]. Also, the different crystalline quality in different samples may cause the opposite results, because we observed a clear (111) peak of Py while they observed a very weak (111) peak. Further investigations are required to clarify the origin of intrinsic contribution in Py films.


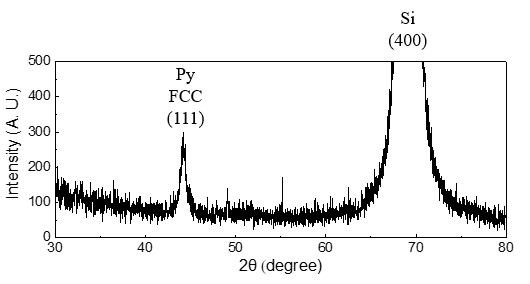


**Fig. S3.** The XRD $\theta-2\theta$ scan spectra of our permalloy sample. A peak at $2\theta\sim44^{\circ}$ indicates the fcc (111) crystalline phase of Py. A large peak at $2\theta\sim70^{\circ}$ arises from the substrate Si (400) [39].

**Note 4. Possible field-induced artefacts**

We checked the possible field-induced artefacts such as mechanical noise or ordinary magnetoresistance. To check this, we prepared non-magnetic Pt (5 nm)/TaO_x_ (1 nm) sample. This sample does not show any AMR but may exhibit other field-induced artefacts if any. Figure S4 shows the transmitted THz pulses for various field orientations (and also for field zero). As shown in the figure, we did not observe any noticeable change by magnetic field in Pt/Ta sample, which implies that the magnetic field-induced mechanical effects do not affect the AMR measurement. This additional experiment can also rule out the possibility of ordinary magnetoresistance effect induced by Lorentz force, which was estimated to be negligibly small (~${4\times10}^{-8}$ % from ${\Delta\rho}/\rho=\mu^{2}B^{2}$, where $\mu(\sim{10}^{-3}m^{2}/V\cdot s)$ is the mobility and $B(=20mT)$ is the external field ) compared to the AMR change (3~4%).


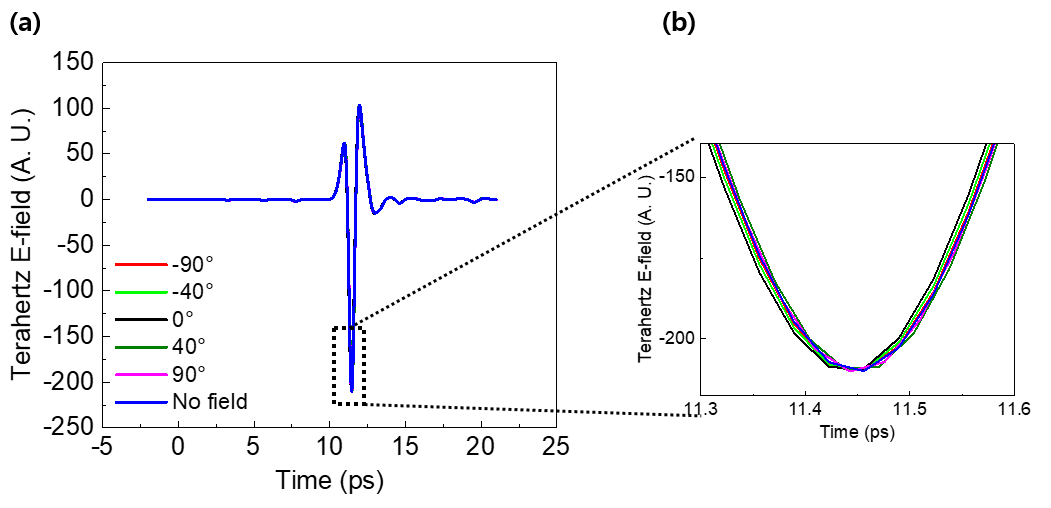


**Fig.S4** (a) Transmitted terahertz pulse depending on the field orientation for Pt (5 nm)/Ta (1 nm) film. Blue curve represents the result without magnetic field. (b) Enlarged pulse peaks.

**Note 5. Fourier transform of time domain pulse**

Figure S5(a) shows the THz pulse, typically obtained in our THz-TDS setup, and Fig. S5(b) is enlarged pulse. The symbols are experimental data that are actually obtained from the machine, and the lines are the guide to the eye. The time interval between neighbouring symbols is 33 *fs*, which corresponds the time resolution of our THz-TDS setup. This means that the theoretical frequency maximum that we can access is about 15 THz (=$1/\left( 2\times33fs \right))$, but in real system the accessible frequency range is reduced due to the noise and large attenuation from the sample. To do the frequency analysis, we perform the Fourier transformation from the time domain pulse data. Fig. S5(c) shows the typical THz spectra obtained from the Fourier transform. As shown in the figure, the meaningful frequency range (FFT amplitude > 0.1) is limited under 4 THz in the absence of sample (The meaningful frequency range is further reduced to under 2 THz when the sample is placed in the THz path (Fig. S5(d))). We note that the THz spectra in our result shows very smooth shape without any dips or peaks. This is important because a dip or peak in spectral amplitude leads to unwanted dip or peak in complex conductivity, which causes a large error in Drude fitting.


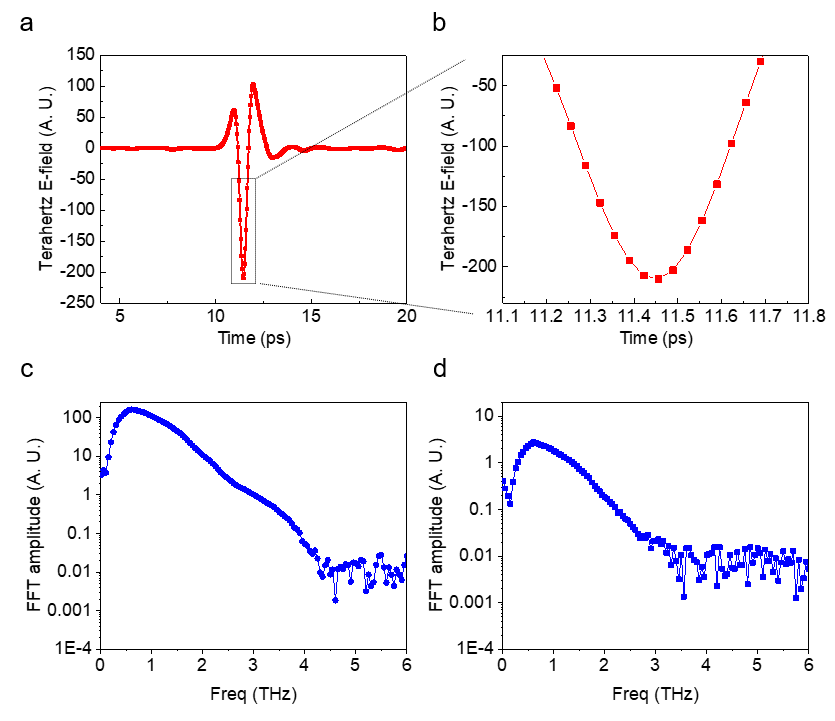


**Fig.S5** (a) Typical time domain THz pulse (b) Enlarged THz pulse. (c) Typical THz spectra obtained by Fourier transform from time domain pulse in the absence of sample (d) THz spectra in the presence of sample.

**References**

35. E. Roman et al., *Phys. Rev. Lett.* **103**, 097203 (2009).

36. S. Lamrani et al., Eur. Phys. *J. Appl. Phys.* **74**, 30302 (2016).

37. DY. Petrovykh et al., *Appl. Phys. Lett.* **73**, 3459 (1998).

38. M. Kateb and S. Ingvarsson, IEEE Sensors Applications Symposium (SAS) pp. 1–5 (2017)

39. J. Keckes., *Acta Materialia* **144**, 862-873 (2018).
